# Vancomycin-induced linear IgA bullous dermatosis mimicking Stevens–Johnson syndrome/toxic epidermal necrolysis treated with etanercept

**DOI:** 10.1016/j.jdcr.2026.04.040

**Published:** 2026-04-29

**Authors:** Rawan Alanazi, Rawan Aldahash, Shada Khalid Alanazi, Sharifah Hussain Almasoud, Nesreen Abdullah Alumair, Walid Alghamdi, Ahmed Alhumidi

**Affiliations:** a College of Medicine, Dar Al Uloom University, Riyadh, Saudi Arabia; b Department of Dermatology, Security Forces Hospital, Riyadh, Saudi Arabia; c College of Medicine, King Faisal University, Al-Ahsa, Saudi Arabia; d College of Medicine, Majmaah University, Majmaah, Riyadh Province, Saudi Arabia; e Department of Pathology, King Saud University, Riyadh, Saudi Arabia

**Keywords:** drug-induced bullous disease, etanercept, linear IgA bullous dermatosis, steroid-sparing therapy, TNF-alpha inhibitor, vancomycin

## Introduction

Linear IgA bullous dermatosis (LABD) is a rare subepidermal autoimmune blistering disorder defined by linear IgA deposition at the basement membrane zone of the skin or mucous membranes.^1^ Drug-induced LABD (DILAD) constitutes a significant proportion of LABD cases, with vancomycin identified as the most common precipitating agent.^1^^,^^2^

We report a case of vancomycin-induced LABD that initially presented with some clinical features concerning for Stevens–Johnson syndrome/toxic epidermal necrolysis (SJS/TEN) and was therefore managed empirically with etanercept prior to diagnostic confirmation.

## Case report

A 62-year-old man with ischemic heart disease requiring an implantable cardioverter-defibrillator, prior ischemic stroke, atrial fibrillation complicated by recent pulmonary embolism and deep venous thrombosis, and a low-risk gastrointestinal stromal tumor was hospitalized for management of an acute ischemic stroke. His hospital course was complicated by severe systemic infection requiring broad-spectrum antimicrobial therapy.

Vancomycin was administered on 3 separate occasions. An initial loading dose and a subsequent 4-day course were tolerated without cutaneous adverse effects. Approximately 2 weeks after completion of the second course, the patient developed a progressive erythematous eruption involving the trunk and extremities. Vancomycin was reinitiated shortly thereafter for treatment of polymicrobial infection (see Table I).

**Table I:** Chronologic summary of vancomycin exposure, cutaneous findings, and management

Date	Vancomycin exposure	Cutaneous findings	Management
October 6	Single loading dose administered	No cutaneous reaction	—
October 16-19	Four-day therapeutic course	No cutaneous reaction	—
November 1	—	Onset of erythematous rash	—
November 3	Vancomycin restarted (after rash onset)	Rash persisting	—
November 5	—	Dermatology consultation	Infusion rate reduced; topical corticosteroids initiated
November 6	Vancomycin discontinued	—	—
November 7	—	Rapid progression with tense bullae and erosions	Punch biopsy performed; etanercept 50 mg (first dose)
November 10	—	Marked clinical improvement	Etanercept 50 mg (second dose)

*BSA*, Body surface area.

A Dermatology consultation was obtained 5 days after rash onset, during which vancomycin was being actively infused. Examination revealed widespread nonblanchable erythematous patches involving the face, trunk, and extremities, without mucosal involvement. Given that the eruption coincided with an ongoing infusion, red man syndrome was initially considered; the infusion rate was reduced, and topical mometasone was initiated. Vancomycin was discontinued the following day.

**Fig 1:**
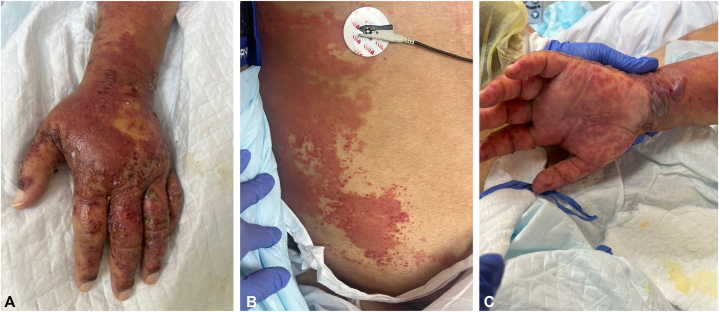
**A, B,** Well-demarcated erythematous plaques with superimposed tense bullae on the trunk and extremities. **C,** Multiple targetoid macules on the palms.

Over the subsequent 24 hours, the eruption progressed rapidly, with the development of tense bullae and superficial erosions involving more than 15% of the body surface area, along with targetoid macules on the palms (Fig 1, *A*–*C*). The ocular, oral, and genital mucosae were spared. Nikolsky and Asboe-Hansen signs were negative, and laboratory evaluation showed no evidence of drug reaction with eosinophilia and systemic symptoms or vasculitis.

Given the rapid progression and extent of epidermal involvement, a severe cutaneous adverse reaction within the Stevens–Johnson syndrome/toxic epidermal necrolysis (SJS/TEN) spectrum was initially suspected. A punch biopsy was obtained, and etanercept (50 mg subcutaneously) was initiated empirically along with clobetasol cream while diagnostic studies were pending. A second dose was administered 3 days later.

**Fig 2:**
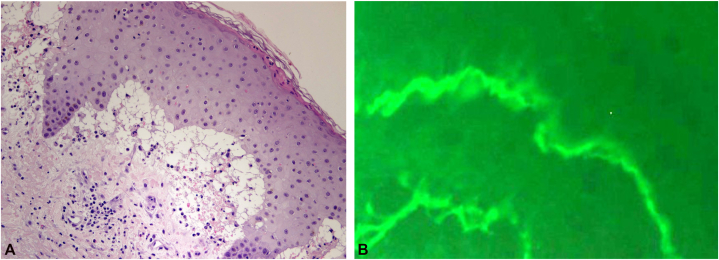
**A,** Hematoxylin and eosin–stained section of a punch biopsy from the left arm showing a subepidermal blister with separation at the dermoepidermal junction and a predominantly neutrophilic infiltrate within the blister cavity (original magnification ×200). **B,** Direct immunofluorescence of perilesional skin demonstrating linear deposition of IgA along the basement membrane zone at the dermoepidermal junction (original magnification ×400).

Following initiation of etanercept, cutaneous erythema began to regress, no new bullae developed, and existing lesions showed early signs of resolution prior to administration of the second dose. After the second dose, continued clinical improvement was observed, with further regression of erythema and healing of existing lesions (Fig 2, *A* and *B*).

**Fig 3:**
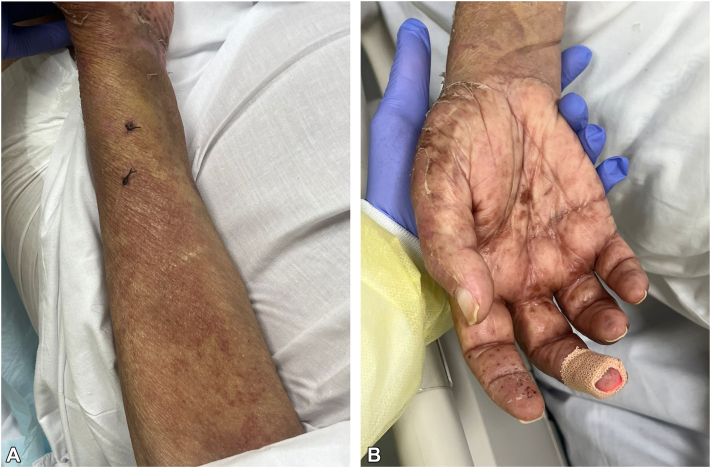
**A, B,** Clinical response following etanercept therapy demonstrating gradual regression of widespread erythema with evolution into post-inflammatory hyperpigmentation within 48 hours of the initial dose.

Given sustained clinical improvement, no additional doses of etanercept were administered. Subsequent histopathologic examination demonstrated a subepidermal blister with neutrophilic infiltration and microabscesses along the dermal–epidermal junction. Direct immunofluorescence (DIF) revealed linear IgA deposition along the basement membrane zone, confirming vancomycin-induced linear IgA bullous dermatosis (Fig 3, *A* and *B*). A timeline of vancomycin exposure, cutaneous findings, diagnostic evaluation, and management is shown in Table I.

## Discussion

Vancomycin-induced linear IgA bullous dermatosis (DILAD) is an uncommon severe cutaneous adverse reaction that may present with rapid blistering and substantial clinical overlap with Stevens–Johnson syndrome/toxic epidermal necrolysis (SJS/TEN).^1^, ^2^, ^3^ This overlap is particularly challenging in critically ill hospitalized patients, where diagnostic uncertainty is common and treatment decisions may be required before histopathologic confirmation.^3^^,^^4^

In this case, abrupt progression to widespread bullae, targetoid lesions, and significant body surface area involvement raised strong initial concern for SJS/TEN. Although the absence of mucosal involvement and a negative Nikolsky sign were atypical, these features do not reliably exclude SJS/TEN early in its course. Definitive differentiation was achieved only after biopsy, with DIF demonstrating subepidermal blistering with linear IgA deposition along the basement membrane zone, confirming DILAD.

Management decisions were therefore guided by the initial working diagnosis of SJS/TEN rather than confirmed DILAD. Despite prompt discontinuation of vancomycin and initiation of topical corticosteroids, the eruption continued to progress. Systemic corticosteroids were avoided because of active severe infection and concern for worsening sepsis. Etanercept was selected as empiric therapy based on its reported efficacy in SJS/TEN and its relatively short half-life,^5^^,^^6^ allowing limited exposure in a medically complex patient. Continued progression after vancomycin withdrawal and rapid stabilization following etanercept initiation suggest that drug cessation and topical therapy alone may not fully account for the observed improvement.

Although etanercept is not an established therapy for DILAD, a biologically plausible mechanism exists. Tumor necrosis factor–α (TNF-α) contributes to neutrophil recruitment and inflammatory amplification at the dermal–epidermal junction, a central feature of IgA-mediated blistering diseases. TNF-α inhibition may therefore attenuate acute inflammatory injury and stabilize disease progression without directly targeting IgA autoantibody production.^7^^,^^8^

Clinical improvement occurred within 48 hours of etanercept initiation, with cessation of new blister formation. Limited prior case reports have described similar use of etanercept in severe DILAD presenting with SJS/TEN–like features.^9^^,^^10^

This case highlights etanercept as an effective therapeutic option for severe, rapidly progressive LABD, particularly when systemic corticosteroids are contraindicated. Its favorable safety profile and biologically plausible mechanism of action support its consideration as a steroid-sparing agent in this setting.

## Conflicts of interest

None disclosed.
